# Targeting *Helicobacter pylori* Through the “Muco-Microbiotic Layer” Lens: The Challenge of Probiotics and Microbiota Nanovesicles

**DOI:** 10.3390/nu17030569

**Published:** 2025-02-03

**Authors:** Olga Maria Manna, Celeste Caruso Bavisotto, Melania Ionelia Gratie, Provvidenza Damiani, Giuseppe Bonaventura, Francesco Cappello, Giovanni Tomasello, Vito D’Andrea

**Affiliations:** 1 Department of Sciences for Promotion of Health and Mother and Child Care, Surgical Pathology Unit, University of Palermo, 90133 Palermo, Italy; olgamaria.manna@unipa.it; 2 Euro-Mediterranean Institute of Science and Technology (IEMEST), 90146 Palermo, Italy; g.melania13@icloud.com (M.I.G.); francesco.cappello@unipa.it (F.C.); 3 Department of Biomedicine, Neurosciences and Advanced Diagnostics (BIND), Institute of Human Anatomy and Histology, University of Palermo, 90127 Palermo, Italy; giuseppe.bonaventura@unipa.it (G.B.); giovanni.tomasello@unipa.it (G.T.); 4 Risk Management and Quality Unit, Hospital University “Paolo Giaccone”, 90127 Palermo, Italy; donatelladamiani@alice.it; 5 Department of Surgery, Sapienza University of Rome, 00161 Rome, Italy; vito.dandrea@uniroma1.it

**Keywords:** gastric mucosa, *Helicobacter pylori*, antibiotic resistance, microbiota, muco-microbiotic layer, nanovesicles, exosomes, probiotics

## Abstract

The muco-microbiotic layer represents a critical biological frontier in gastroenterology, emphasizing the intricate interplay between the protective mucus, its resident microbiota, and extracellular vesicles. This review explores the functional morphology of the gastric mucosa, focusing on the gastric muco-microbiotic layer, its role as a protective barrier, and its dynamic interaction with some of the most insidious pathogens such as *Helicobacter pylori* (*H. pylori*). Highlighting the multifaceted mechanisms of *H. pylori* pathogenesis, we have delved into bacterial virulence factors, host immune responses, and the microbiota’s regulatory effects. Novel therapeutic strategies for *H. pylori* eradication, including traditional antibiotic therapies and emerging adjuvant treatments like probiotics and probiotic-derived extracellular vesicles, are critically examined. These findings underscore the potential of targeting nanovesicular interactions in the gastric mucosa, proposing a paradigm shift in the management of *H. pylori* infections to improve patient outcomes while mitigating antibiotic resistance.

## 1. Introduction

The interaction between gastric mucus, its associated microbiota, and the related nanovesicles, referred to as the “muco-microbiotic layer” [1,2,3,4], plays a pivotal role in understanding gastric pathophysiology and therapeutic strategies also against *Helicobacter pylori* (*H. pylori*). The significance of this layer lies above all in its protective barrier function and its communication (i.e., crosstalk) with human cells via extracellular vesicles. This work examines the role of such interactions in the pathogenesis of *H. pylori* infections and proposes a novel therapeutic perspective: the use of probiotics and, mostly, their extracellular vesicles to enhance treatment efficacy and reduce associated side effects. This approach could not only improve infection management but also help mitigate the rising issue of antibiotic resistance.

## 2. A Brief View on Gastric Structure and Muco-Microbiotic Layer

The stomach structure is made up of multiple layers: mucosa, submucosa, muscularis propria, and serosa. The mucosa, the innermost layer, is essential for secreting important digestive factors and for protecting the stomach itself from hydrochloric acid (HCl) [5].

Particularly, the epithelium of the gastric mucosa forms glands (gastric glands, of various types), and it is composed of a single layer of columnar epithelial cells, which, among other things, play a key role in the secretion of mucus. This mucus forms a protective barrier against the highly acidic environment created by the secretion of HCl by parietal cells. In addition, the epithelial cells are tightly bound by junctional complexes that prevent gastric acid from penetrating deeper layers of the stomach. This glandular epithelium also presents gastric pits that contain various types of secretory cells, among which are the already mentioned parietal cells (which secrete HCl); the chief cells (which produce pepsinogen); and other cytotypes, including undifferentiated (“stem”) elements. [6].

Indeed, the epithelium is involved in maintaining the mucosal barrier. This barrier is supported by the continuous renewal of epithelial cells (by mitosis and the differentiation of these undifferentiated elements), which are replaced roughly every 4–7 days, ensuring the mucosa’s resilience to physical and chemical stressors. When the epithelium is damaged or its protective function impaired, conditions such as gastritis, peptic ulcers, or cancer can develop [7].

The structural integrity and function of the gastric epithelium are therefore fundamental to maintaining stomach health. Disruptions in this epithelium, such as in the case of *H. pylori* infection, can lead to alterations in the mucosal barrier, contributing to digestive disorders [8].

**Figure 1:**
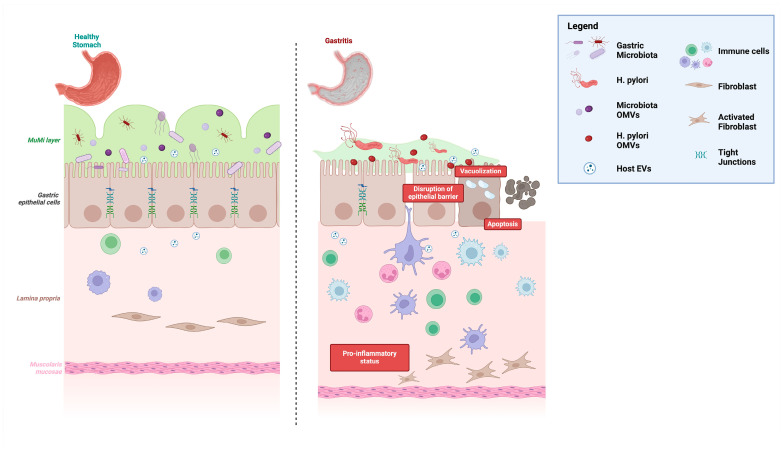
Schematic representation of the impact of *H. pylori* infection on the gastric mucosa.

In the last years, we proposed a new vision of the entire wall of the gastrointestinal apparatus by introducing the “muco-microbiotic layer” concept (see Figure 1), i.e., the fact the in vivo the innermost layer of the stomach and the bowel is constituted by (1) the mucus, which creates the microenvironment to support (2) the microbiota, which cross-talks with human cells by (3) nanovesicles, i.e., outer membrane vesicles for the bacterial component and exosomes for the human counterpart [1,2,3,4]. However, in common histological preparations, after treatments of the fresh tissues (e.g., biopsies) with alcoholic solutions, this layer “disappears” since its main constituent (the mucus) is soluble in alcohol.

In our opinion, since the knowledge of the structure (histology) of an organ lays the basis for the comprehension of its pathophysiology, ignoring such a fundamental component for the physiology of the organ alters the possibility of fully understanding the mechanisms underlying the onset of diseases of the same and in turn compromises the identification of new therapeutic strategies. With this in mind, here we summarize the main information related to *H. pylori* gastritis and its therapy, introducing some new elements in this story: the gastric muco-microbiotic layer and, in particular, its nanovesicles, a tool for crosstalk between human and bacterial cells.

## 3. *H. pylori* Infection Pathogenesis: Clues and Remarks

*H. pylori* is a ubiquitous pathogen that infects approximately 50% of the global population. The prevalence of *H. pylori* infection varies considerably between developed and developing nations, with rates estimated at 30% to 50% in the former and 85% to 95% in the latter [9,10]. The prevalence of *H. pylori* varies across Europe, being lower in the northern and western regions compared to eastern and southern Europe. Many studies indicate a significant decline in *H. pylori* prevalence, with an average annual reduction of 3.1% [11,12]. It is noteworthy that *H. pylori* prevalence tends to increase with age but levels off in individuals around 50 years and older, especially in high-prevalence areas. Northern Europe has the lowest infection rates, while Eastern and Southern Europe exhibit the highest rates, reaching up to 84% in countries such as Portugal and Poland [13,14].

*H. pylori* is a gram-negative, spiral-shaped bacterium belonging to the *Helicobacteraceae* family, discovered by Barry Marshall and Robin Warren in 1980 [15]. This microorganism predominantly infects the mucous layer of the stomach, utilizing its capability to flourish in acidic environments by neutralizing local acidity through the production of urease and other factors [16,17]. Although the exact transmission mode is still largely uncertain, it is generally believed to occur primarily through oral–fecal, gastric–oral, or oral–oral routes, often via ingesting contaminated food and water, with lower socioeconomic status being a significant risk factor [18,19]. Factors contributing to infection include inadequate hygiene practices; the consumption of contaminated food or water; and certain dietary habits, particularly those rich in milk, meat, and fried foods [20,21]. Antibiotic resistance poses a significant challenge to treatment, as the overuse of antibiotics has led to increased resistance rates [22,23]. Infection is usually contracted in childhood, with only about 20–30% of those infected exhibiting symptoms, resulting in many cases being asymptomatic. As a result, undiagnosed infections may lead to chronic inflammation, such as non-atrophic and potentially atrophic gastritis. Clinically, chronic gastritis has frequently been linked to the development of gastric cancer, including adenocarcinomas and MALT lymphomas [24].

*H. pylori* infection pathogenesis involves bacterial virulence factors, host immune responses, and environmental conditions. There are some key components that contribute to the development of clinical conditions such as gastritis and ulcers in *H. pylori* infection [25]. First, the urease activity of *H. pylori* plays a crucial role in neutralizing the acidic environment of the stomach. In particular, *H. pylori* possesses an acid adaptation mechanism that enables it to thrive in the stomach’s harsh acidic environment by regulating urease activity [26]. The urease gene cluster comprises seven genes responsible for producing urease and the activity of accessory proteins such as UreE and HypA, which facilitate the nickel transfer necessary for activating urease. This enzyme is essential for the bacterium’s acid resistance, allowing urea to enter only under acidic conditions and preventing alkalization [27,28]. Notably, *H. pylori* generates significant amounts of ammonium derived from urea and may even expel NH3/NH4 to neutralize incoming protons, thereby linking acid resistance to nitrogen metabolism [29]. Moreover, urease modulates interactions with macrophages, influencing phagosome pH and megasome formation, thus enhancing *H. pylori’s* survival against the innate immune response [30]. The second key component consists of the bacterium’s flagella-mediated motility, which enables it to move toward the host’s gastric epithelial cells. This movement is followed by the interaction of bacterial adhesins with host cell receptors, resulting in successful colonization and persistent infection. Finally, *H. pylori* releases various effector proteins and toxins, including cytotoxin-associated gene A (Cag A) and vacuolating cytotoxin A (VacA), which lead to host tissue damage [31].

The immune response to *H. pylori* infection involves a complex interplay between bacterial factors and the host’s immune system, resulting in chronic gastric inflammation. Initially, *H. pylori* triggers an innate immune response [32]. This activation subsequently initiates an adaptive immune response, wherein CD4+ T-helper cells promote a Th1 response while B cells generate antibodies against the bacterium. However, *H. pylori* employs various immune evasion strategies to elude detection and signaling by both T and B cells, ultimately leading to chronic inflammation, tissue damage, and increased turnover of gastric epithelial cells, which can cause ulcers and potentially predispose individuals to gastric cancer [33,34]. In addition, *H. pylori* affects mucin production in the gastric mucosa, influencing the protective barrier and its colonizing ability. This modulation of mucin production allows the bacterium to establish persistent infections [35]. Understanding these interactions between the immune system and *H. pylori* is crucial for developing effective therapies and prevention strategies for diseases associated with this pathogen.

## 4. Conventional Therapy for *H. pylori* Eradication: A Brief Overview

Many studies have explored various pharmacological treatments for *H. pylori*-induced gastritis, focusing on enhancing eradication rates and addressing antibiotic resistance. Progress made in the management of *H. pylori* infection was covered in the sixth edition of the Maastricht/Florence 2021 Consensus Report [36], in which recommendations are provided based on the best available evidence and relevance to the management of *H. pylori* infection in various clinical fields.

Very in brief, the most employed therapies seem to be Dual Therapy with Proton Pump Inhibitors (PPIs) and amoxicillin. High-dose PPI combined with amoxicillin has been revisited as a treatment option. While some studies report this regimen as cost-effective and safe, others indicate that its success rates may not be consistently acceptable [37].

Rifabutin-Based Triple Therapy: For patients with refractory *H. pylori* infections, rifabutin-based triple therapy has shown effectiveness against multi-drug-resistant strains, offering a viable alternative when standard treatments fail [38].

Quadruple Therapy: Bismuth-based quadruple therapy, which includes a PPI, bismuth, tetracycline, and metronidazole, is recommended as an initial treatment, especially in regions with high antibiotic resistance [39].

Noteworthily, tetracycline, as part of quadruple therapy, is not available in many countries, including some EU ones [40] This has been highlighted as a problem in the clinical practice. However, a meta-analysis proposed to use doxycycline instead of tetracycline as an effective treatment [41].

Main pros and cons of these therapies are listed in Table 1.

Emerging Therapies: Research into novel therapeutic regimens is ongoing, with studies examining the efficacy of different antibiotic combinations and adjunctive therapies to improve treatment outcomes. Among adjuvants, the use of probiotics is currently under investigation [42]. Indeed, the management of *H. pylori*-induced gastritis is evolving, with recent studies supporting the use of both established and emerging supplementations. Treatment selection should consider local antibiotic resistance patterns, patient history, and the potential for adverse effects. In the following paragraph, we will discuss the use of probiotic supplementation in *H. Pylori* eradication.

## 5. Probiotics in the Supplementation of *H. pylori* Therapy: A Novel Strategy

*H. pylori* infection remains one of the most prevalent global health issues, contributing to chronic gastritis, peptic ulcers, and gastric cancer. The growing antibiotic resistance has necessitated exploring complementary treatments like probiotics to enhance eradication success rates and mitigate side effects. In this brief paragraph, we summarize the results of some very recent clinical studies and meta-analyses on the use of probiotics as adjunctive therapy for the eradication of *H. pylori*. The report delves into their efficacy, safety, mechanisms of action, and influence on microbiota diversity while also outlining advancements in therapeutic strategies.

Ivashkin et al. [43] conducted a randomized controlled trial demonstrating that the incorporation of *Limosilactobacillus reuteri* DSM 17648 into standard therapy significantly increased eradication success to 96.7% compared to 86% in placebo. The probiotic also reduced gastrointestinal discomfort associated with therapy. Zhang et al. [44] evaluated *Saccharomyces boulardii* CNCM I-745 combined with triple therapy. This approach resulted in improved microbiota profiles and alleviated gastrointestinal symptoms, with one subgroup achieving a 93.18% eradication rate. Ivashkin et al. [43] and Zhang et al. [44] both highlighted how probiotic adjuncts counteract common challenges like diarrhea, nausea, and abdominal pain, providing a safer alternative for patients undergoing traditional antibiotic regimens.

Li et al. [45] conducted a meta-analysis revealing an increased eradication rate from *Lactobacillus reuteri* BBC3 supplementation. This also translated to lower incidences of side effects, highlighting the dual therapeutic benefits. Wang et al. [46] identified microbial shifts following eradication treatment, including an expansion of probiotics like *Leuconostoc mesenteroides*, which further suppressed *H. pylori*. Peng et al. [47] investigated oral microbiota modulation through dual therapy (vonoprazan–amoxicillin) combined with probiotics, and their results showed notable recovery of oral microbiota diversity post-eradication.

Lu et al. [48], in an umbrella meta-analysis, identified that probiotic addition improved both eradication rates and gastrointestinal health metrics. This supports the prophylactic and therapeutic promises of probiotics. Wang et al. [49], in a network meta-analysis, established *Bifidobacterium*- and *Lactobacillus*-based mixtures as highly effective adjuvants. Probiotic combinations like *Bifidobacterium*–*Lactobacillus*–*Saccharomyces* achieved eradication rates up to 88.2%, underscoring the enhanced potential of multi-strain approaches. He et al. [50] reported smaller fluctuations in gastric microbiota among patients treated with probiotic-enhanced therapies, emphasizing a stabilizing effect on the overall gut microbiome during therapy. FitzGerald et al. [51] explored how multi-strain probiotics exhibited strain-level proliferation during therapy, reinforcing their direct impact on eradication efficacy and gastrointestinal health. Finally, in a randomized, placebo-controlled trial, Viazis et al. demonstrated that the integration of probiotics into a 10-day concomitant non-bismuth quadruple therapy regimen for *H. pylori* significantly enhances the eradication rate and concurrently diminishes the incidence of adverse effects [52].

These studies, as also schematically presented in Table 2, emphasize the integration of probiotics into *H. pylori* treatment as a transformative step in therapeutic strategies. Key outcomes include: (1) enhanced eradication rates with targeted probiotics, (2) the stabilization and recovery of microbiota diversity across gastric and intestinal landscapes, (3) reduced antibiotic-related side effects, and (4) promising results from multi-strain probiotic formulations that improve overall patient outcomes.

While these findings establish probiotics as an effective adjunct, further investigations are warranted to fine-tune strain-specific protocols and optimize therapeutic combinations. Particularly, it is necessary to unveil the role of nanovesicles in the crosstalk between microbiota and gastric cells during treatment with probiotics.

## 6. Probiotic Nanovesicles: A Possible Ally in *H. pylori* Eradication Strategies?

In recent years, increasing attention has been directed toward postbiotic molecules and non-viable components, such as cell-free supernatants (CFS) and extracellular vesicles (EVs) derived from gut microorganisms, for their role in the interplay between gut flora and human tissues, influencing the gut mucosa and overall host health. Emerging studies have highlighted that EVs from gut microbiota can influence inflammatory processes by modulating the production of pro-inflammatory cytokines [53]. *H. pylori* produces EVs, also named outer membrane vesicles (OMVs) that serve as highly effective vehicles for delivering virulence factors to host cells, contributing to the establishment of a chronic inflammatory environment within the gastric mucosa [54,55].

EVs are nanosized, membrane-bound particles, ranging from 20 to 300 nm in diameter [56]. EVs are actively released by nearly all cell types into a wide range of biological fluids, such as blood, urine, saliva, and cerebrospinal fluid. These vesicles serve as intercellular communication mediators, carrying diverse cargo, including proteins, lipids, RNA, and DNA, which reflect the physiological or pathological state of their originating cells. Their ubiquitous presence and functional versatility make EVs crucial players in both normal biological processes and disease mechanisms [57,58,59,60].

During infection numerous pathogen bacteria release OMVs that contribute to the dissemination of pathogenic components (e.g., pathogen-associated molecular patterns (PAMPs) including, proteins, lipids, nucleic acids, and carbohydrates) to the surrounding cells and tissues [60,61,62]. The OMVs produced by *H. pylori* were derived from its outer membrane, like many other Gram-negative bacteria. These vesicles, ranging in size from 20 to 450 nm, play a pivotal role in facilitating communication both among bacteria and with their surrounding environment, making them key contributors to bacterial pathogenesis [53]. Studies have confirmed that these *H. pylori*-derived OMVs retain a variety of surface molecules characteristic of the parent bacterium, including lipopolysaccharides (LPS), peptidoglycan, and a phospholipid and protein composition similar to that of the outer membrane [63,64]. These surface proteins of *H. pylori*-derived OMVs, including adhesins and lipopolysaccharides, play a crucial role in enhancing their interaction with gastric epithelial cells, thereby increasing their immunogenic potential. Additionally, they encapsulate cytoplasmic components such as translation-associated proteins and virulence factors [65,66]. Key virulence-associated proteins, including urease subunits, VacA, CagA, and the adhesins BabA and SabA, have been identified within these vesicles. CagA, for instance, disrupts cellular signaling pathways upon delivery into gastric epithelial cells, leading to cytoskeletal rearrangements and pro-inflammatory signaling cascades. It appears to promote the disruption of tight junctions between epithelial cells [67].

VacA, on the other hand, can induce vacuole formation in host cells, contributing to cellular stress and further promoting an inflammatory environment [68]. LPS stimulates Toll-like receptors on host immune cells, triggering the release of pro-inflammatory cytokines such as IL-8 and TNF-α. Moreover, *H. pylori*-OMVs also carry γ-glutamyl transpeptidase, HtrA protease, GroEL, catalase, and various metabolic and ribosomal proteins, highlighting their complexity and functional versatility in bacterial pathogenesis. Collectively, these molecules contribute to a chronic inflammatory state within the gastric mucosa, undermining epithelial integrity and playing a significant role in gastric adenocarcinoma progression [69,70].

In contrast, the beneficial effects of EVs produced by probiotic bacteria, such as *Lactobacillus*, *Bifidobacterium*, and *Saccharomyces* species, are well-documented. These microorganisms, which can colonize the human gut, release EVs enriched with bioactive compounds, including lipoteichoic acids, polysaccharides, metabolites, and small RNAs [71]. These vesicles play a significant role in modulating the host’s immune system by promoting anti-inflammatory responses, enhancing mucosal barrier integrity, and supporting the maintenance of gut homeostasis [72,73].

Probiotic EVs are similarly nanosized, generally ranging between 50 and 300 nm in diameter, and share structural similarities with other bacterial EVs, having a bilayer lipid membrane that confers stability and enables efficient molecular transfer [74]. However, their biochemical profiles differ markedly from those of pathogenic EVs. Probiotic EVs are enriched in molecules with anti-inflammatory and cytoprotective properties, such as lipoteichoic acid (LTA), polysaccharides, small RNAs, and metabolites including short-chain fatty acids (SCFAs) [75]. These vesicles are thought to play an immunomodulatory role, reducing inflammatory signaling pathways and enhancing mucosal barrier integrity. For example, metabolites contained within probiotic EVs have been shown to influence epithelial cell metabolism, promoting the maintenance of tight junction proteins and reducing epithelial permeability [76]. Their ability to influence immune signaling pathways highlights their therapeutic potential in mitigating inflammation and restoring gut health [3,77].

Several studies have demonstrated the anti-inflammatory and antimicrobial properties of *Lactobacillus* strains. Wang et al. [78] conducted a study involving 78 patients with *H. pylori* infection who were treated with specific *Lactobacillus* strains. The results showed that these probiotic strains significantly alleviated gastrointestinal discomfort and reduced gastric inflammation in *H. pylori*-infected patients, highlighting their therapeutic potential in managing *H. pylori*-associated gastritis (Table 3). Another study by Wang et al. [79] investigated the inhibitory effects of six stomach-derived *Lactobacillus* species (*L. oris* F0423, *L. delbrueckii* LMG P-21905, *L. crispatus* CCFM1118, *L. salivarius* SP2, and *L. gasseri* CIP 110105) on *H. pylori* growth. Among these, *L. crispatus* CCFM1118 demonstrated exceptional strength, showing the ability to succeed in highly acidic conditions and in environments with elevated bile salt concentrations (Table 3).

Few studies have evaluated the effects of EVs derived from probiotics on *H. pylori*-induced gastritis. One such study investigated the ability of EVs and CFS derived from the *L. crispatus* strain RIGLD-1 to mitigate inflammatory responses triggered by *H. pylori* in vitro, using AGS human gastric epithelial cells as a model. The results demonstrated that both EVs and CFS effectively suppressed inflammatory signaling pathways activated by *H. pylori*, likely through the downregulation of NF-κB and MAPK pathways, which play a central role in mediating immune responses in gastric epithelial cells [80] (Table 3). *L. gasseri* CFS has a protective effect on the gastric mucosa, as it has been demonstrated to reduce autophagy induced by *H. pylori* EVs. The beneficial effects of *Lactobacillus* spp. are attributed to the presence of bioactive compounds in their CFS, including organic acids, bacteriocins, and potentially EVs. These bioactive substances not only exhibit antimicrobial activity against pathogenic bacteria but also appear to modulate autophagy processes, offering promising therapeutic benefits in managing conditions associated with *H. pylori*-induced autophagy dysregulation [54] (Table 3).

This crosstalk between pathogenic and probiotic vesicles is hypothesized to modulate the gastric microenvironment by promoting a shift towards a less inflammatory state. Probiotic EVs may neutralize or mitigate the harmful effects of *H. pylori* vesicles, thereby contributing to the re-establishment of a balanced immune response and reinforcing the protective mucus layer of the gastrointestinal tract, particularly in the stomach and intestines. This suggests their potential as adjuvant therapies in managing *H. pylori*-related gastritis. The interplay between these vesicular populations could reshape the gastric mucosal environment, enhancing the resilience of the mucosal barrier, improving clinical outcomes, and paving the way for innovative therapeutic strategies (Figure 1).

On the left, the image depicts a healthy gastric mucosa with an intact epithelial barrier and a continuous muco-microbial (MuMi) layer. This layer consists of protective mucus and a diverse gastric microbiota populated by numerous beneficial bacterial species, which together maintain mucosal integrity and contribute to homeostasis. On the right, the gastric mucosa colonized by *H. pylori* is shown. *H. pylori* competes with beneficial bacterial species, reducing microbial diversity and compromising the protective MuMi layer. This disruption leads to epithelial barrier damage, chronic inflammation, and an impaired mucosal environment. Probiotic interventions, through the release of outer membrane vesicles (OMVs) and other bioactive metabolites, counteract these effects by mitigating inflammation, restoring epithelial integrity, and promoting the re-establishment of the MuMi layer, ultimately supporting gastric mucosal homeostasis.

## 7. Conclusions

This review aimed to illustrate the pivotal role of the gastric muco-microbiotic layer, and in particular of one of its main components, i.e., the nanovesicles, in maintaining gastrointestinal health and to highlight its susceptibility to disruption by pathogenic microorganisms like *H. pylori*. The integration of probiotics and their extracellular vesicles as therapeutic agents emerges as a promising avenue to enhance eradication rates, mitigate inflammation, and stabilize microbiota diversity.

Moreover, integrating insights into the structure and function of the gastric muco-microbiotic layer holds significant implications for surgical research, particularly in the realms of pathophysiology and surgical semeiotics. For surgeons, understanding the composition and dynamic interactions among gastric mucus, associated microbiota, and extracellular vesicles offers a novel perspective in managing gastric pathologies such as peptic ulcers, chronic gastritis, and gastric tumors.

The disruption of this biological barrier is a pivotal factor in the onset and progression of conditions often necessitating surgical intervention. Surgical semeiotics can benefit from a heightened focus on these microstructural mechanisms, enhancing diagnostic precision through the identification of markers indicative of barrier–microbiota dysfunction. Furthermore, these advancements may inform innovative perioperative strategies, including the use of probiotics or extracellular vesicle-targeted therapies, to optimize postoperative recovery and mitigate inflammatory complications.

Future research should focus on the functional characterization of probiotic-derived vesicles and their interplay with pathogenic counterparts to optimize therapeutic protocols. These advancements could pave the way for novel, targeted interventions that address both *H. pylori* pathogenesis and broader gastrointestinal health challenges.

## Figures and Tables

**Table 1:** A summary of main pros and cons of conventional therapies, according to the Maastricht/Florence 2021 Consensus Report [36].

Employed Therapy	Drugs	PROS	CONS	Ref.
**Dual Therapy**	PPIs and Amoxicillin	Cost-effective and safe	Its success rates may be low.	[37]
**Triple Therapy**	PPIs, Amoxicillin, and Rifabutin	A viable alternative when dual treatments fail	Its success rates may be low as well as dual therapy.	[38]
**Quadruple Therapy**	PPI, Bismuth, Tetracycline, and Metronidazole	The success rate is higher than the dual and triple therapies	The patient has difficulty tolerating it due to the side effects and often stops the therapy before the end.	[42]

**Table 2:** Schematic view of main results of most significant randomized controlled trials and meta-analyses presented in paragraph 5.

Type of Study	Examined Probiotics	Main Results	Ref.
Randomized controlled trial	Limosilactobacillus reuteri DSM 17648	Probiotics: (1) alleviated symptoms due to therapy, and (2) permitted a very high eradication rate.	[43]
Randomized controlled trial	Saccharomyces boulardii	Probiotics: (1) alleviated symptoms due to therapy, and (2) in one subgroup permitted a very high eradication rate.	[44]
Meta-analysis	Lactobacillus reuteri BBC3	Probiotics (1) alleviated symptoms due to therapy, and (2) increased significantly the eradication rate.	[45]
Meta-analysis	Various, including also Lactobacillus, Saccharomyces boulardii, and Bifidobacterium	Probiotic addition improved both eradication rates and gastrointestinal health metrics.	[48]
Meta-analysis	Bifidobacterium- and Lactobacillus-based mixtures	Probiotics multi-strain approaches enhanced potential of eradication.	[49]
Randomized controlled trial	Bifidobacterium-based mixture	Probiotics modulate gastrointestinal microbiota also after *H. Pylori* eradication.	[50]
Randomized controlled trial	Lactobacillus-based mixtures	Multi-strain probiotics have a direct impact on eradication efficacy and gastrointestinal health.	[51]
Randomized controlled trial	Combination of combined four probiotic strains, i.e., Lactobacillus Acidophilus, Lactiplantibacillus plantarum CCFM8610, Bifidobacterium lactis BS01, and Saccharomyces boulardii	Probiotic supplementation (1) significantly enhances the eradication rate and (2) diminishes the incidence of adverse effects in quadruple therapies.	

**Table 3:** Summary of findings from in vitro and clinical studies investigating the effects of probiotics and EVs on *H. pylori*-associated inflammation. In vitro studies highlight the role of EVs and CFS from Lactobacillus species in mitigating inflammatory signaling and modulating autophagy induced by *H. pylori*. Clinical trials further support the therapeutic potential of probiotics in reducing gastric inflammation and alleviating symptoms associated with *H. pylori* infection.

Study Type	Experimental System	Key Findings	Ref.
Clinical study	*H. pylori*-infected patients	Probiotic treatment with Lactobacillus strains reduced gastric inflammation and gastrointestinal discomfort.	[78]
In vitro	Stomach-derived Lactobacillus species (*L. oris* F0423, *L. delbrueckii* LMG P-21905, *L. crispatus* CCFM1118, *L. salivarius* SP2, and *L. gasseri* CIP 110105)	*L. crispatus* CCFM1118 inhibited *H. pylori* growth even in highly acidic and bile salt-rich environments.	[79]
In vitro	AGS human gastric epithelial cells	EVs and CFS from *L. crispatus* RIGLD-1 suppressed inflammatory signaling (NF-κB, MAPK pathways) triggered by *H. pylori.*	[80]
In vitro	AGS cells treated with *L. gasseri* CFS	CFS reduced H. pylori EV-induced autophagy, highlighting protective effects on gastric mucosa.	[54]

## Data Availability

Not applicable.

## Abbreviations

The following abbreviations are used in this manuscript:

Cag A CFS	Cytotoxin-associated gene A Cell-free supernatants
EVs	Extracellular vesicles
HCl	Hydrochloric acid
*H. pylori*	Helicobacter pylori
LPS	Lipopolysaccharides
LTA	Lipoteichoic acid
OMVs	Outer membrane vesicles
PAMPs	Pathogen-associated molecular patterns
PPIs	Proton Pump Inhibitors
SCFAs	Short-chain fatty acids
VacA	Vacuolating cytotoxin A
